# Analysis of Knowledge and Satisfaction in Virtual Clinical Simulation among Nursing Students: A Mixed Study

**DOI:** 10.3390/nursrep14020081

**Published:** 2024-04-27

**Authors:** Daniel Medel, Mercedes Reguant, Tània Cemeli, María Jiménez Herrera, Carme Campoy, Aida Bonet, Montserrat Sanromà-Ortíz, Judith Roca

**Affiliations:** 1 Department of Nursing and Physiotherapy, Faculty of Nursing and Physiotherapy, University of Lleida, 2 Montserrat Roig, 25199 Lleida, Spain; daniel.medel@udl.cat (D.M.); carme.campoy@udl.cat (C.C.); aida.bonet@udl.cat (A.B.); montserrat.sanroma@udl.cat (M.S.-O.); 2 Department of Research Methods and Diagnosis in Education, University of Barcelona, 08007 Barcelona, Spain; mreguant@ub.edu; 3 Department of Nursing, University Rovira Virgili, 43002 Tarragona, Spain; maria.jimenez@urv.cat; 4 Health Education, Nursing, Sustainability and Innovation Research Group (GREISI), 25199 Lleida, Spain

**Keywords:** learning, nursing student, satisfaction, virtual simulation, simulation training, knowledge

## Abstract

Virtual simulation offers a powerful educational tool with considerable, albeit underexplored potential. This technology immerses students in lifelike digital scenarios, fostering the acquisition of knowledge and skills necessary for their future careers. This study aimed to assess knowledge acquisition and satisfaction outcomes among students using a virtual simulation teaching approach. The specific objectives were (1) to compare pre-and posttest knowledge acquisition, (2) to investigate the influence of prior professional experience on knowledge, and (3) to explore satisfaction levels with virtual simulation. One hundred and fifty-nine nursing students participated in a virtual simulation-based clinical intervention, entailing the resolution of a virtual adult patient hospitalized with respiratory pathology. Sociodemographic data and prior professional experience were collected, and knowledge was evaluated through pre-to-post tests. Satisfaction levels were assessed using open-ended questions. Quantitative data were analyzed using descriptive statistics, Wilcoxon, Mann–Whitney U, and Cohen’s tests, while qualitative data underwent keyword-in-context analysis. Significant differences were noted between pre- and posttest knowledge levels, with prior experience showing no significant impact on knowledge acquisition. Participants reported high levels of satisfaction. Lexicometric analysis identified four clusters of words related to the key terms “simulation”, “learn”, “activity”, and “knowledge”. Virtual clinical simulation effectively enhances knowledge acquisition and fosters satisfaction, with students recognizing the positive impact of this approach on their learning. Consequently, virtual simulation contributes to the training of competent health professionals.

## 1. Introduction

Clinical simulation is an indispensable teaching component in professional health training [1,2]. Specifically, virtual simulation entails a partially immersive experience, faithfully reconstructing reality within the confines of a computer screen [3]. This learning experience enables nursing students to develop motor control, decision-making, and communication skills [4]. While still relatively underexplored, virtual simulation has garnered attention as a compelling pedagogical resource [5], fostering interactive online realms that imbue learners with a sense of contextual presence [6]. Within this framework, various technologies have emerged to facilitate virtual simulations. Notable examples include virtual reality systems equipped with interactive glasses that enable students to actively engage with simulated environments [7], computer-based platforms tailored for case resolution [5,8,9,10] to enhance clinical decision-making skills [9,11], and the utilization of virtual task trainers for practicing technical procedures [12].

Furthermore, it is worth noting that the onset of the COVID-19 pandemic precipitated a shift in conventional educational paradigms [13]. This global health crisis mandated an expeditious transition from in-person instructional methodologies to online modalities, with virtual learning supplanting erstwhile in-person clinical practice settings [13,14,15,16,17]. Virtual clinical simulation, in particular, emerged as a salient solution, freeing education from the constraints of temporal and spatial limitations. This modality facilitates concurrent training for larger cohorts of students [18], unbounded by geographical confines or physical presence requirements [19]. Notably, virtual simulation offers the flexibility of asynchronous [20] and individualized learning experiences [5]. Moreover, this technology allows for synchronous simulation activities across disparate geographical locations worldwide [9]. Furthermore, virtual simulation platforms enable students to confront complex scenarios that may be logistically challenging to organize in traditional face-to-face settings [5,21]. 

Studies conducted using this methodology [5,22] agree that students participating in these simulations demonstrate superior theoretical and practical knowledge development compared to those receiving traditional training. Knowledge forms the cornerstone of nursing education, underpinning the professional competence of future nurses [23]. Virtual simulations thus emerge as indispensable tools, enabling students to interact with virtual patients or other health professionals to address clinical scenarios [24]. These immersive scenarios compel students to analyze information, assess diverse options, and make informed decisions—all while developing their communication and teamwork acumen as well as knowledge and cognitive skills essential for their future careers [25].

Another noteworthy aspect of virtual simulation is that students often express higher satisfaction with their learning and training, reducing fear and anxiety about their future profession [26,27]. This satisfaction stems from clinical simulation, allowing students to practice in a safe and controlled environment without risking patient safety [28,29]. Satisfaction is experienced by the fulfilment of needs through actions [30], which in simulation training creates a positive emotional state among students who find contentment in their learning experiences. This sensation also has a positive impact on their academic performance and personal growth by boosting motivation, self-confidence, and participation [31]. Additionally, virtual clinical simulations tend to be interactive and engaging, fostering greater student involvement in their learning [5,13,19,32].

Virtual clinical simulation also affords institutions the opportunity to diversify the array of clinical scenarios accessible to students [5]. While it does not seek to wholly replace face-to-face clinical simulation, it is a supplementary teaching approach [11,33]. Indeed, certain facets of face-to-face clinical simulation pose challenges in virtual replication. Nonetheless, the emergence of virtual simulation constitutes a seminal advancement in nursing education, underscoring the imperative of its integration into comprehensive simulation programs [24].

Thus, the integration of virtual simulation into nursing curricula appears promising, yet further research is needed to substantiate its efficacy [3]. Accordingly, we created a classroom-based virtual simulation activity on respiratory pathology to assess students’ knowledge and satisfaction outcomes engaging with this tool. This aimed to ascertain the outcomes in terms of knowledge and satisfaction that students achieve through virtual simulation and whether prior professional experience in health influences these outcomes. Consequently, the primary hypothesis posits that nursing students exposed to the virtual simulation teaching–learning strategy demonstrate knowledge acquisition and high satisfaction levels. The secondary hypothesis anticipates that irrespective of prior professional experience, nursing students achieve comparable knowledge levels about respiratory pathology after completing virtual simulation, owing to its immersive learning opportunities. Therefore, the principal objective of this study was to assess the outcomes (knowledge and satisfaction) obtained by students using the virtual simulation teaching approach. The specific objectives encompassed (1) quantifying knowledge pre- and post-virtual simulation through a comparative pre- and posttest, (2) evaluating the potential impact of previous professional experience on virtual simulation-related knowledge, and (3) exploring students’ satisfaction with the virtual tool.

## 2. Materials and Methods

### 2.1. Study Design

A convergent mixed study in a parallel format was adopted [34], facilitating the concurrent collection and analysis of quantitative (QUAN) and qualitative (QUAL) data to enrich findings and elucidate conclusions [35]. 

### 2.2. Context and Participants

This study was conducted among second-year nursing degree students at the Faculty of Nursing and Physiotherapy (FIF), University of Lleida (UdL), focusing on the subject of Adult Nursing Care (6 European Credit Transfer and Accumulation System (ECTS) (150 h)). The Nursing Degree program in Spain consists of 240 ECTS, equivalent to approximately 6000 h of study, distributed across 4 academic years. 

The inclusion criteria for participants were as follows. All students must have been enrolled for the entire subject, while those who did not attend or did not provide consent for data sharing due to enrollment or personal reasons (health concerns or force majeure) were excluded. The final participants totaled 159, with 15 students excluded from the group.

Participants were thoroughly informed about the objectives of the study, and their consent was obtained before data collection, a responsibility undertaken by the teaching team. The virtual simulation teaching experience, based on the creation of an interactive clinical case, constituted an obligatory component of the Adult Nursing Care 2 course within the second year of the nursing degree program. Students who opted not to consent to data collection and analysis were excluded from the study. Moreover, participants were apprised of the voluntary nature of data disclosure, and clear instructions on how to opt out were provided, with assurances of no detrimental repercussions. Importantly, participants were offered no incentives or rewards in exchange for their involvement. Subsequently, data anonymization was executed via codes generated from IP in strict adherence to university regulations. This study was approved by the Research and Transfer Ethics Committee (CERT) of the University of Lleida.

### 2.3. Virtual Simulation Learning Experience

The research team developed a virtual simulation interactive case featuring a hospitalized patient with respiratory pathology. Embedded in the video were educational capsules that were strategically integrated to rectify or reinforce knowledge pertinent to the case at hand. This case formation constitutes an integral component of a broader project known as SAVI (Simulación AudioVisual Interactiva). Comprising an interdisciplinary ensemble, the creation team encompasses two nurses, a physician, a biomedical scientist, and a computer engineer, collectively possessing expertise in both healthcare and pedagogy, with specialized proficiency in clinical simulation and digital content creation.

High-fidelity audiovisual content featuring standardized patients and health professionals was recorded at the 4dHealth simulation center “https://4dhealth.com (accessed on 1 January 2024)” for heightened realism. Once edited, the content was uploaded to a web platform where students interacted with the case during the activity, offering multiple development options based on their clinical judgment.

The virtual simulation sessions were conducted in face-to-face classroom seminars, each with 15–20 students, and lasted two hours. A total of 8 seminars were conducted.

### 2.4. Instruments and Data Collection

The data collection instrument was created ad hoc, comprising three sections: (1) basic sociodemographic data such as age, gender, nursing school entry point, prior professional experience in health, and prior clinical simulation experience; (2) a knowledge test consisting of 10 single-choice questions assessing theoretical and practical aspects related to the clinical case, with four alternative responses, only one of which was correct without penalty for incorrect responses. The maximum score was 10, and the minimum was 0 (Table 1). The final section included (3) an overall satisfaction score regarding the activity, accompanied by an open question prompting respondents to justify their satisfaction rating on a scale ranging from 1 (very low) to 10 (very high).

Students completed the questionnaire in person or online via the Virtual Campus platform. The baseline knowledge pretest and collection of sociodemographic data were conducted during the prebriefing session, with the posttest and satisfaction assessment administered at the beginning of the debriefing.

Regarding the knowledge test (Table 1), a maximum total response time was set at 8 min, equating to 48 s per question. Before data collection, a pilot test involving 10 students was conducted to evaluate the time taken and comprehension of the questions and answers, with no necessary adjustments. Data were collected from February to April 2023.

### 2.5. Data Analysis

Normality tests (Kolmogorov–Smirnov) revealed that none of the variables (pretest, posttest, satisfaction level) followed a normal distribution (*p* < 0.001). Therefore, non-parametric tests were used for the analysis. Descriptive statistics (mean, standard deviation, percentages), Wilcoxon test for related samples (pre- and posttest), and Mann–Whitney U test for unrelated samples (comparisons between students with and without professional experience) were used. Additionally, Cohen’s test was used to analyze the effect size. Quantitative data were analyzed using SPSS version 25, and the significance level was set at *p* < 0.05.

Responses to the open-ended question underwent qualitative analysis using keywords-in-context (KWIC) documentary analysis (22). This method identifies keywords and uses the context to understand the underlying meaning. The lexicometric analysis was facilitated by the software IRaMuTeQ 0.7 alpha 2 2020. This free, R-based software offers various statistical techniques for analyzing textual data. Additionally, researchers searched the text for keywords and identified descriptive phrases to gain deeper insights into the meaning of the data.

## 3. Results

### 3.1. Description of Participants

A total of 159 nursing students aged between 19 and 43 years, with a mean age of 20.87 years (SD = 3.46), participated in the study. Most (77.4%) were female, and a high school diploma was the primary entry point to nursing school (71.1%). Most students (74.8%) had no prior professional experience in the health field, although all had previous training simulation experience (159 of 159). Table 2 shows the sample characteristics.

### 3.2. Quantitative Assessment of Knowledge and Satisfaction

Significant differences were observed between pre- and posttest results (Table 3). In the posttest, they exhibited a higher mean score (7.60 out of 10) and a lower standard deviation (1.33). A moderate effect size (0.66) was also observed, along with a moderate correlation between both scores. Students’ satisfaction perception toward virtual simulation was high, with a mean score of 8.84 out of 10 (SD = 0.88).

As shown in Table 4, previous professional experience in the health field did not affect the results (pretest, posttest, satisfaction). There were no statistically significant differences when comparing the outcomes between the students with and without prior health experience. Regarding the effect of previous work experience, values lower than 0.20 indicate no effect. It should be noted that the value is negative regarding satisfaction level, indicating that the perception of satisfaction of the group without experience was higher. 

### 3.3. Qualitative Assessment of Satisfaction

**Figure 1:**
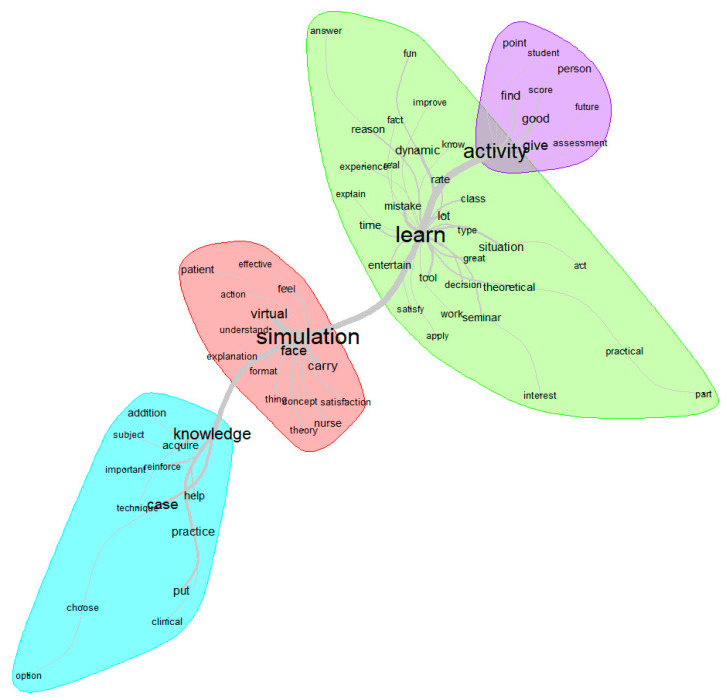
Similarity analysis on students’ perceived satisfaction in virtual simulation.

Figure 1 provides a graphical overview of the lexicometric discourse on satisfaction perceived by the students in the virtual simulation and the latent meaning in their responses. Four clusters of meaning were identified around the terms most frequently used by the students (keywords). These clusters represent four themes: “simulation” (frequency of occurrence 126), “learn” (frequency 80), “activity” (frequency 104), and “knowledge” (frequency 66). Table 5 summarizes the analysis by keyword, providing their description and examples of verbatim responses including related terms.

## 4. Discussion

The findings from this study indicate the effectiveness of virtual simulation in enhancing learning outcomes, as demonstrated by a significant increase in posttest scores alongside high satisfaction levels among students. The moderate correlation between pretest and posttest scores indicates that knowledge accrues after simulation. These findings align with extant research [5,17,22,36,37], delineating that virtual clinical simulation can significantly augment theoretical knowledge and practical skills. Notably, Zaragoza-García et al. [13] observed an augmentation in knowledge acquisition across 15 out of 15 studies scrutinizing the impact of virtual simulation. Moreover, the umbrella review by Cant et al. [38] corroborates these findings, elucidating the positive ramifications on both knowledge and skill acquisition.

Regarding satisfaction, various studies [5,16,25,32,39] highlight consistently high satisfaction levels among participants. A study by Lubbers and Rossman [40] describes students’ perception of virtual simulation as engaging and enjoyable, while Cant and Cooper [19] emphasized its interactive, stimulating, and enjoyable nature. 

Students’ previous professional experience in health care did not affect the final knowledge outcomes, suggesting that virtual simulation benefits learners at all experience levels. This indicates that simulation can benefit both students and experienced healthcare professionals, thereby serving as a valuable tool for continuing education, skills maintenance, and healthcare training [36,40]. However, it is worth noting that the success of the simulation experience hinges on the balance between the learning challenge and the student’s skills [41].

In the results of the lexicometric analysis on satisfaction, the most frequently used terms forming the nodes were “simulation”, “learn”, “activity”, and “knowledge”. The analysis of each node and its related words revealed several insights. For instance, elements within the node “simulation” suggest that students appreciated the broad array of concepts, theories, explanations, and understandings simulation provides them in an engaging and interactive manner. They believe that these explanations helped them better understand the concepts and identify the causes of their errors, which is in line with the findings by Cant and Cooper [19]. Additionally, the words “nurse” and “patient” appear, demonstrating the impact of simulation on nursing care quality and, thus, enhancing patient safety [5,42]. 

Within the “learn” node, virtual simulation can serve as a continuum and bridge between academic learning and practical care, echoing findings from prior research [42,43] that emphasize its role in bridging the gap between theory and real-world practice. This gap is defined as a mismatch between what nursing students are taught in academic settings and what they experience in clinical settings [44]. According to this node, students, recognizing the dynamic and enriching nature of the learning experience facilitated by virtual simulations, value its active, motivating, and enjoyable characteristics, which correlate with their elevated satisfaction scores. Similarly, Cant et al. [38] elaborate on students’ positive reception of virtual simulations, deeming them accessible, enjoyable, and engaging learning modalities. Notably, all these findings fully agree with the study by Goldsworthy et al. [20], demonstrating that virtual simulations enhance learning, increase confidence, and improve students’ ability to prioritize.

Within the “activity” node, the evaluative aspects of simulation emerge. Simulation as a competency assessment offers an effective way to measure learning and skill development in a relevant practical context that resembles real-life situations [45]. Among the evaluative aspects, students establish a direct link between scores and errors, highlighting again the importance of error-based learning in a secure environment to better prepare for real-world patient care [10]. This also relates to future professional development, as shown in studies such as Bogossian et al. [22], which link e-simulation to both augmented knowledge and clinical performance. Other authors, such as Yang et al. [33], advocate for the broader application of virtual clinical simulation-based interventions, positing benefits not only in bolstering general knowledge but also in catering to the specialized needs of newly graduated nurses, especially in specialized care units. Moreover, simulation addresses practice-based learning, facilitating the management of negative emotions such as fear, refinement of clinical skills, and the fortification of nurse–patient relationships [46]. Nevertheless, nurse educators face the challenge of integrating these methodologies and teaching strategies, such as virtual simulation, into curricula to ensure that the knowledge and skills learned during virtual simulations can be applied in clinical contexts with patient-centered care [38]. 

Lastly, in the “knowledge” node, the related terms describe the potential of virtual simulation for developing clinical cases as an essential pedagogical tool and aid for clinical practice. Students appreciate how the resource assists them in acquiring new knowledge, both theoretical and practical, and in consolidating previously acquired knowledge. Notably, the presented simulation contains training capsules, thus offering direct hints and comments for assistance [47]. Furthermore, students mention terms in this cluster, such as “option” or “choose”, indicating decision making. Lapum et al. [48], Goldsworthy et al. [20], and Jans et al. [25] regard decision making as a fundamental cognitive skill in nursing, one that can be honed through virtual simulation. In addition, nursing decision making is a critical process integrated into the daily routines of nurses. This process entails assessing information, identifying health issues, establishing care objectives, and selecting appropriate interventions to address patient health concerns [49,50].

Finally, it is noteworthy that these results are encouraging and suggest that virtual simulation is a valuable tool for nursing education, as it enables comprehensive learning. It is important to continue using virtual simulation as a pedagogical tool and to conduct further research to assess its impact on learning.

### 4.1. Implications for Practice

This study underscores the importance of nursing or health sciences education through clinical simulation. It provides valuable insights for educators seeking to incorporate new teaching strategies into the classroom, complementing traditional teaching approaches. Virtual simulation in the classroom is a valuable, adaptable, and cost-effective educational resource [51]. Furthermore, it offers the possibility of repeating simulations and working on content in a personalized manner [10] without the need to be physically present in a specific space [52]. As technology advances, virtual simulation becomes more realistic and sophisticated and offers more learning opportunities. Therefore, this technology is likely poised to become an increasingly important component of education and training programs for students and professionals in health sciences. 

### 4.2. Limitations

As possible limitations, it is noted that this study was conducted in a single group without a control group (n = 159). Additionally, there is the issue of the carry-over effect in the results, as there was no washout period between the pretest intervention and posttest [53] or the potential for response bias [25]. In light of the results presented, similar studies are suggested with quasi-experimental designs, including a control group and multicenter approaches. Longitudinal assessment is also recommended to evaluate knowledge retention over time [47]. Furthermore, there is a lack of evidence to determine which type of simulation is most effective [54]. Finally, in this learning experience, debriefing was conducted, but its impact on satisfaction or knowledge acquisition was not evaluated, since the knowledge test was administered at the beginning of the session. 

## 5. Conclusions

Virtual clinical simulation emerges as a classroom pedagogical strategy that enhances students’ knowledge while being perceived as a tool that generates high satisfaction. Likewise, the analysis of satisfaction reveals terms that positively reinforce the perceived benefits, such as “simulation”, “activity”, “learn”, and “knowledge”. This strategy is transferable to other fields within health sciences and professional training. All of this aims to contribute to the development of more competent professionals, thus having a direct impact on society by improving safety and quality in patient care.

## Figures and Tables

**Table 1:** Multiple-choice questionnaire linked to the virtual case.

Question	Answers
(1) The FVC for a patient with an obstructive pattern is:	* (a) Normal (b) Very reduced (c) Reduced (d) I don’t know
(2) Values above 45 mmHg of PaCO indicate:	(a) Hypoxemia * (b) Hypercapnia (c) Respiratory failure (d) I don’t know
(3) In high-flow oxygen therapy systems, which statement is false?	(a) It regulates FiO2 precisely (b) The Venturi system is not high flow * (c) It depends on the patient’s respiratory pattern (d) I don’t know
(4) Regarding the Monaghan mask, which statement is true?	* (a) It is a low-flow device (b) Oxygen enters the attached reservoir and allows intermittent flow (c) It is most indicated for cases of hypercapnia (d) I don’t know
(5) Renal function due to prerenal cause can be affected in a patient with:	* (a) Acute kidney injury (AKI) (b) Renal tumor (c) Renal lithiasis (d) I don’t know
(6) Dysarthria is:	* (a) Difficulty articulating sounds or speech disorder (b) Excessive sweating (c) Joint pain and immobility (d) I don’t know
(7) Regarding creatinine, which statement is false?	(a) It comes from protein catabolism (b) It is eliminated in urine * (c) Higher creatinine means higher glomerular filtration (d) I don’t know
(8) Amlodipine is a drug:	* (a) Calcium antagonist, regulates blood pressure (b) Bronchodilator (c) Immunosuppressant (d) I don’t know
(9) Which of these substances has sympathomimetic effects?	* (a) Salbutamol (b) Methylprednisolone (c) Ipratropium bromide (d) I don’t know
(10) What substance allows rapid recovery of blood glucose in case of hypoglycemia?	* (a) 50% Glucose (b) Glucagon (c) Insulin (d) I don’t know

* The asterisk indicates the correct answer.

**Table 2:** Sample characteristics of the number (N) and frequencies (%).

Variables		N	%
Age *		20.87	3.46
Sex	Men	36	22.6
	Women	123	77.4
Entry point to nursing school	High school diploma	113	71.1
	Training courses	35	22
	University degrees	5	3.1
	Over 25–45 years old	6	3.8
Health worker	No	119	74.8
	Yes	40	25.2

* Mean and standard deviation (SD).

**Table 3:** Pretest and posttest differences in knowledge.

	N	Mean	SD	Wilcoxon Text Sig. Asint. Bil	Effect Size (r)	Pearson Correlation Sig. Asint. Bil
Pretest	159	4.80	1.78	<0.001	0.66	0.381 **
Postest	159	7.60	1.33			

** The correlation is significant at level 0.01 (two-tailed).

**Table 4:** Differences between students with and without professional experience in healthcare.

	Pretest	Postest	Satisfaction Level
Mann–Whitney U	2059.500	2319.500	2034.000
Wilcoxon	2879.500	3139.500	9055.000
Z	−1.290	−0.247	−1.358
Sig. Asint. (bilateral)	0.197	0.805	0.174
R	0.13	0.07	−0.11

**Table 5:** Qualitative analysis synthesis.

Keyword	Description Related to Satisfaction	Related Words	Related Word Verbatims
Simulation	Refers to satisfaction regarding the pedagogical resource in terms of usability.	-Aspects of virtual simulation source: “nurse”, “patient”, “format”, “virtual”, and “action” -Pedagogical aspects: “concept”, “theory”, “understand”, and “explanation” -Impact of the dynamic: “satisfaction”, “effective”, and “feel”	*“...great satisfaction thanks to the wealth of knowledge provided throughout the execution and its originality”. P155* *“...the explanations help you understand why you failed the question or provide additional theoretical information”. P18* *“...it was a very dynamic and effective way to acquire new knowledge in a dynamic and fun way”. P36*
Learn	Addresses satisfaction with the experience of the teaching–learning process.	-Active and motivating learning experience: “real”, “fun”, “interest”, “dynamic”, “great”, “entertain”, “satisfy”, and “improve” -Learning actions: “mistake”, “decision”, “experience”, “situation”, “act”, “apply”, “answer”, and “explain” -Context elements: “theoretical”, “practical”, “seminars”, and “class”	*“…a very realistic tool, and it’s very well designed to enhance learning”. P35* *“…very comprehensive, both for evaluating graphics and analytics, and being able to grasp your mistake and continue advancing. I consider it a highly practical tool to motivate further learning”. P109* *“It’s an alternative and dynamic way that helps you make decisions”. P3*
Activity	Satisfaction is valued in terms of achievement and professional development.	-Evaluative aspects: “score”, “assessment”, and “point” -Resource transfer: “persons”, “students”, and “future” -Utility: “good” and “ find”	*“…solving practical cases so that both in clinical practice and in future work, we can deliver the best care to patients and handle any situation”. P49* *“It allowed me to learn from my mistakes so as not to repeat them in the future”. P150*
Knowledge	Evaluates satisfaction concerning knowledge and skills acquired at both theoretical and practical levels.	-Potential of the tool for theoretical and practical learning: “choose”, “option”, “acquire”, “practice”, “reinforce”, “addition”, “important”, “help”, “technique”, “practice”, and “clinical”	*“It represents an alternative way to face clinical scenarios and at the same time influence them, but from a more external point of view, without experiencing any pressure. Personally, this implies enhanced learning”. P122* *“…as reinforcement, it’s very good because theoretical explanations were integrated dynamically”. P10*

## Data Availability

Please contact the corresponding author (tania.cemeli@udl.cat) to request data.

## Public Involvement Statement

No public involvement in any aspect of this research.

## Guidelines and Standards Statement

This manuscript was drafted against the MMR-RHS checklist (Reporting mixed methods research in rehabilitation and health science) [55] for mixed research.
